# Supplementary material for: Evaluating the Diagnostic Performance of Hemoglobin in the Diagnosis of Iron Deficiency Anemia in High-Altitude Populations: A Scoping Review
Source: Int J Environ Res Public Health. 2023 Jun 13;20(12):6117. doi: 10.3390/ijerph20126117 (PMC10297848; doi:10.3390/ijerph20126117)
Supplement: Supplementary file 1 [file ijerph-20-06117-s001.zip › ijerph-2182272-supplementary.pdf]

Supplementary Material

Supplementary material 1. Search strategy

The detailed search strategy for each database can be viewed below

Filter: None

Database	Search strategy (03/05/22)	Search
**PubMed**	**#1 Altitude:**<br>(Altitud*[Title/Abstract] OR Mountain*[Title/Abstract] OR Andean*[Title/Abstract] OR highland*[Title/Abstract] OR Altitudes[MeSH Terms] OR “Meters above sea level”[Title/Abstract] OR "MASL"[Title/Abstract] OR "MAMSL"[Title/Abstract])<br>**#2 Anemia for Iron-Deficiency:**<br>("anemia/diagnosis"[MeSH Terms] OR "anemia, iron deficiency/diagnosis"[MeSH Terms] OR "anemia*"[Title/Abstract] OR "anaemia*"[Title/Abstract] OR "hypoferritinemia*"[Title/Abstract] OR "iron*"[Title/Abstract])<br>**#3 Hemoglobin:**<br>("hemoglobins"[MeSH Terms] OR "hemoglobin*"[Title/Abstract] OR "haemoglobin*"[Title/Abstract] OR "ferrohemoglobin"[Title/Abstract] OR "ferrohaemoglobin"[Title/Abstract])<br>**#4 Other hematological parameters of iron status:**<br>("ferritin*"[Title/Abstract] OR "isoferritin*"[Title/Abstract] OR "transferrin*"[Title/Abstract] OR "Transferrin-Binding Proteins"[Title/Abstract] OR "Hepcidin*"[Title/Abstract] OR "erythropoietin*"[Title/Abstract] OR "TfR"[Title/Abstract] OR "sTfR"[Title/Abstract] OR “TFR-F"[Title/Abstract] OR “Iron status”[Title/Abstract])	114
**Scopus**	**#1 Altitude:**<br>TITLE-ABS-KEY (Altitud* OR Mountain* OR Andean* OR highland* OR Altitudes OR “Meters above sea level” OR "MASL" OR "MAMSL")<br>**#2 Anemia for Iron-Deficiency:**<br>TITLE-ABS-KEY ("anemia*" OR "anaemia*" OR "hypoferritinemia*" OR "iron*")<br>**#3 Hemoglobin:**<br>TITLE-ABS-KEY ("hemoglobins" OR "hemoglobin*" OR "haemoglobin*" OR "ferrohemoglobin" OR "ferrohaemoglobin")<br>**#4 Other hematological parameters of iron status:**<br>TITLE-ABS-KEY ("ferritin*" OR "isoferritin*" OR "transferrin*" OR "Transferrin-Binding Proteins" OR "Hepcidin*" OR "erythropoietin*" OR "TfR" OR "sTfR" OR “TFR-F" OR “Iron status”)	196
**WoS/Core collection**	**#1 Altitude:**<br>TS = (Altitud* OR Mountain* OR Andean* OR highland* OR Altitudes OR “Meters above sea level” OR "MASL" OR "MAMSL")<br>**#2 Anemia for Iron-Deficiency:**<br>TS = ("anemia*" OR "anaemia*" OR "hypoferritinemia*" OR "iron*")<br>**#3 Hemoglobin:**<br>TS = ("hemoglobins" OR "hemoglobin*" OR "haemoglobin*" OR "ferrohemoglobin" OR "ferrohaemoglobin")<br>**#4 Other hematological parameters of iron status:**<br>TS = ("ferritin*" OR "isoferritin*" OR "transferrin*" OR "Transferrin-Binding Proteins" OR "Hepcidin*" OR "erythropoietin*" OR "TfR" OR "sTfR" OR “TFR-F" OR “Iron status”)	139
**Embase**	**#1 Altitude:**<br>(altitud*:ti,ab OR mountain*:ti,ab OR andean*:ti,ab OR highland*:ti,ab OR altitudes OR 'meters above sea level':ti,ab OR masl:ti,ab OR mamsl:ti,ab)<br>**#2 Anemia:**<br>('anemia'/exp OR anemia OR 'anemia, iron deficiency/diagnosis' OR anemia*:ti,ab OR anaemia*:ti,ab OR hypoferritinemia*:ti,ab OR iron*:ti,ab)<br>**#3 Hemoglobin:**<br>('hemoglobins'/exp OR hemoglobin*:ti,ab OR haemoglobin*:ti,ab OR ferrohemoglobin:ti,ab OR ferrohaemoglobin:ti,ab)<br>**#4 Other hematological parameters of iron status:**<br>('ferritin'/exp OR 'ferritin' OR 'isoferritin'/exp OR 'isoferritin' OR 'transferrin'/exp OR 'transferrin' OR 'transferrin-binding proteins':ti,ab OR 'hepcidin'/exp OR 'hepcidin' OR 'erythropoietin'/exp OR 'erythropoietin' OR 'tfr':ti,ab OR 'stfr':ti,ab OR 'tfr-f':ti,ab OR 'iron status':ti,ab)	175

**COCHRANE LIBRARY CENTRAL**	**#1 Altitude:**<br>(Altitud*:ti,ab OR Mountain*:ti,ab OR Andean*:ti,ab OR highland*:ti,ab OR [mh Altitudes] OR "Meters above sea level":ti,ab OR MASL:ti,ab OR MAMSL:ti,ab)<br>**#2 Anemia:**<br>([mh anemia] OR [mh "anemia, iron deficiency"] OR anemia*:ti,ab OR anaemia*:ti,ab OR hypoferritinemia*:ti,ab OR iron*:ti,ab)<br>**#3 Hemoglobin:**<br>([mh hemoglobins] OR hemoglobin*:ti,ab OR haemoglobin*:ti,ab OR ferrohemoglobin:ti,ab OR ferrohaemoglobin:ti,ab)<br>**#4 Other hematological parameters of iron status:**<br>(ferritin*:ti,ab OR isoferritin*:ti,ab OR transferrin*:ti,ab OR "Transferrin-Binding Proteins":ti,ab OR Hepcidin*:ti,ab OR (erythropoietin*OR NEXT ""TfR""):ti,ab OR sTfR:ti,ab OR TFR-F:ti,ab OR "Iron status":ti,ab)	18
**LILACS by virtual health library**	**#1 Altitude:**<br>(Altitude* OR Highland* OR Andes*)<br>**#2 Anemia:**<br>(anemia* OR anaemia*)<br>**#3 Hemoglobin:**<br>(Hemoglobin* OR Haemoglobin*)<br>**#4 Other hematological parameters of iron status:**<br>(Ferritin* OR Transferrin* OR Hepcidin* OR erythropoietin*)	11
**Ovid / Medline**	**#1 Altitude:**<br>(Altitud* or Mountain* or Andean* or highland*).tw. or exp Altitudes/ or "Meters above sea level".tw. or MASL.tw. or MAMSL.tw.)<br>**#2 Anemia:**<br>(exp anemia/ or exp "anemia, iron deficiency"/ or anemia*.tw. or anaemia*.tw. or hypoferritinemia*.tw. or iron*.tw.)<br>**#3 Hemoglobin:**<br>(exp hemoglobins/ or hemoglobin*.tw. or haemoglobin*.tw. or ferrohemoglobin.tw. or ferrohaemoglobin.tw.)<br>**#4 Other hematological parameters of iron status:**<br>ferritin*.tw. OR isoferritin*.tw. OR transferrin*.tw. OR "Transferrin-Binding Proteins".tw. OR Hepcidin*.tw. OR "erythropoietin*OR "TfR"".tw. OR sTfR.tw. OR TFR-F.tw. OR "Iron status".tw.	91

Supplementary material 2. Reasons for exclusion of studies

Author-year	Title	Exclusion reasons
Hofvander Y. et al. 1968	Hematological investiga tions in Ethiopia with special reference to a high iron intake.	Not full text
Piedras et al. 1985	Sensitivity and specificity of erythrocyte indices in the diagnosis of iron deficiency in infants and women living at 3 different altitudes	Not full text
Sánchez et al. 1985	Seric iron and transferrin: Comparative study in mothers and newborn at high altitude and sea level	Not full text
Coulibaly et al. 1987	Modifications of hemato-biological parameters in pregnant women in a migrating population in northern Cameroon: prevalence of anemia, iron and folates deficiencies	Not full text
Estrella et al. 1987	Evaluation of iron-deficiency anemia by an iron supplementation trial in children living at a 2,800-m altitude.	Not full text
Yepez et al. 1987	Iron status in Ecuadorian pregnant women living at 2,800 m altitude: relationship with infant iron status.	Not full text
Hammerlund et al. 1989	Hematocrit, hemoglobin and iron status of north American College. Females living at an elevation of 2183m	Not full text
Téllez et al. 1994	Circulating proteins and iron status in blood as indicators of the nutritional status of 10- to 12-year-old Bolivian boys	Not full text
Yepez et al. 1994	[High altitude anemia: validity of definition criteria].	Not full text
Diez-Ewald et al. 1997	Prevalence of anemia, iron, folic acid and vitamin B12 deficiency in two Bari Indian communities from western Venezuela.	Not full text
León-Velarde et al. 2000	Hematological parameters in high altitude residents living at 4,355, 4,660, and 5,500 meters above sea level	Not full text
Lin et al. 2003	Iron status and effect of early iron supplementation on sub-clinical iron deficiency in rural school-age children from mountainous areas of Beijing.	Not full text
Idris et al. 2005	Iron deficiency anemia in moderate to severely anemic patients	Not full text
Ragip et al. 2009	A reference range for pregnant women living at high altitude	Not full text
Brothers et al. 2010	Hematological and Physiological Adaptations Following 46 Weeks of Moderate Altitude Residence	Not full text
Xi et al. 2018	Serum hepcidin level and erythropoietin in elderly male tibetans and Han residents at different altitudes	Not full text
Curtain et al. 1965	Haptoglobins and transferrins in Melanesia: Relation to hemoglobin, serum haptoglobin and serum iron levels in population groups in Papua—New Guinea	Wrong outcome
Tafari et al. 1972	“Physiologic anaemia” of infancy at high altitude	Wrong outcome
Franzetti S et al. 1984	Body iron reserves of rural and urban Guatemalan women of reproductive age.	Wrong outcome
Tufts et al. 1985	Distribution of hemoglobin and functional consequences of anemia in adult males at high altitude.	Wrong outcome
Dirren et al. 1994	Altitude correction for hemoglobin	Wrong outcome
Makeshova et al. 2004	Characteristics of erythropoiesis regulation in population living at high altitude.	Wrong outcome
Castillo Bohorquez M. et al. 2009	Detección de deficiencias subclínicas de hierro a partir del índice receptor soluble de transferrina-ferritina en niños sanos de 1 a 10 años de edad residentes en alturas de 300 y 2600 msnm	Wrong outcome
Osei A. et al. 2010	Nutritional status of primary schoolchildren in Garhwali Himalayan villages of India	Wrong outcome
Paredes et al. 2012	Ferritina sérica en mujeres de 15 - 30 años a nivel del mar y en la altura	Wrong outcome
Hurtado et al. 2013	Niveles de hemoglobina en pacientes en hemodiálisis a nivel del mar y a mayor altitud, y su relación con la calidad de vida	Wrong outcome
Lundgrin et al. 2013	Plasma hepcidin of Ethiopian highlanders with steady-state hypoxia	Wrong outcome
Harvey-Leeson et al. 2016	Anemia and Micronutrient Status of Women of Childbearing Age and Children 6-59 Months in the Democratic Republic of the Congo.	Wrong outcome
Akomo et al. 2017	Soya, maize and sorghum ready-to-use therapeutic foods are more effective in correcting anaemia and iron deficiency than the standard ready-to-use therapeutic food: randomized controlled trial.	Wrong outcome
Allen A. et al. 2017	Iron status and anaemia in Sri Lankan secondary school children: A cross-sectional survey	Wrong outcome
Orces et al. 2017	Prevalence of anemia among older adults residing in the coastal and andes mountains in Ecuador: Results of the sabe survey	Wrong outcome
Ozarda et al. 2017	A nationwide multicentre study in Turkey for establishing reference intervals of haematological parameters with novel use of a panel of whole blood.	Wrong outcome
Ocas-Córdova et al. 2018	Hemoglobin Concentration in Children at Different Altitudes in Peru: Proposal for [Hb] Correction for Altitude to Diagnose Anemia and Polycythemia	Wrong outcome
Salah et al. 2018	The prevalence and predictors of iron deficiency anemia among rural infants in nablus governorate	Wrong outcome
Arkhestova et al. 2019	Incidence rate of latent iron deficiency in children aged one year living in different climatic and geographical zones of the Kabardino-Balkar Republic	Wrong outcome
Choque-Quispe et al. 2019	Proportion of anemia attributable to iron deficiency in high-altitude infant populations	Wrong outcome
Sharma et al. 2019	Reexamination of hemoglobin adjustments to define anemia: altitude and smoking	Wrong outcome
Yanamandra et al. 2019	Erythropoietin and ferritin response in native highlanders aged 4-19 years from the Leh-Ladakh region of India	Wrong outcome
Goodrich et al. 2020	The importance of lean mass and iron deficiency when comparing hemoglobin mass in male and female athletic groups.	Wrong outcome
Orsango et al. 2020	Efficacy of processed amaranth-containing bread compared to maize bread on hemoglobin, anemia and iron deficiency anemia prevalence among two-to-five year-old anemic children in Southern Ethiopia: A cluster	Wrong outcome

	randomized controlled trial.	
Staub K. et al. 2020	La concentración de hemoglobina de hombres jóvenes en altitudes residenciales entre 200 y 2000 m refleja la topografía de Suiza	Wrong outcome
Yu et al. 2020	Iron deficiency is a possible risk factor causing right heart failure in Tibetan children living in high altitude area.	Wrong outcome
Breenfeldt et al. 2021	Effects of altitude and recombinant human erythropoietin on iron metabolism: a randomized controlled trial.	Wrong outcome
Figueroa-Mujica et al. 2022	A Critical Analysis of the Automated Hematology Assessment in Pregnant Women at Low and at High Altitude: Association between Red Blood Cells, Platelet Parameters, and Iron Status	Wrong outcome
Ford et al. 2022	Factors associated with anaemia among adolescent boys and girls 10-19 years old in Nepal.	Wrong outcome
Cook et al. 2005	The influence of high-altitude living on body iron	Wrong population
El Menchawy et al. 2015	Efficacy of Multiple Micronutrients Fortified Milk Consumption on Iron Nutritional Status in Moroccan Schoolchildren.	Wrong population
Chandyo et al. 2016	The prevalence of anemia and iron deficiency is more common in breastfed infants than their mothers in Bhaktapur, Nepal.	Wrong population
Donahue et al. 2017	Prevalence of Iron Deficiency and Iron Deficiency Anemia in the Northern and Southern Provinces of Rwanda.	Wrong population
Locks et al. 2019	Changes in growth, anaemia, and iron deficiency among children aged 6-23 months in two districts in Nepal that were part of the post-pilot scale-up of an integrated infant and young child feeding and micronutrient powder intervention	Wrong population
Orsango et al. 2021	Iron deficiency anemia among children aged 2-5 years in southern Ethiopia: a community-based cross-sectional study.	Wrong population
Gebreegziabher et al. 2011	Iron status of women from selected rural areas of Sidama zone, southern Ethiopia	Wrong publication type
Casimiro et al. 2013	Growth attainment and hematological status of low-income preschool children of metropolitan guatemala city	Wrong publication type
Trompetero et al. 2015	Behavior of the indicators of the erythropoiesis and the state of the iron in colombian university population at different heights	Wrong publication type
Varma et al. 2015	Hematological parameters and erythropoiesis regulation of true native highlanders	Wrong publication type
Robalino et al. 2016	Comparison of Hemoglobin Concentration Adjusted for Altitude and Serum Iron and Ferritin to Diagnose Anemia in Childhood in Highlands	Wrong publication type
Gaffer et al. 2017	Complete Blood Count in high altitude area 2500 meters above sea level among Saudi adults : a cross sectional hospital based study from October 2016 to December 2016.	Wrong publication type
McClung et al. 2018	Prolonged High Altitude Exposure Results in Elevated Erythroferrone and Diminished Hepcidin Levels in Healthy Young Male Volunteers	Wrong publication type
Roman-Sanchez et al. 2020	Association between dietary iron intake and levels of serum ferritin and hemoglobin in adult young women living in highlands	Wrong publication type

Supplementary material 3.

Figure S1: Heat map of the countries of the included studies

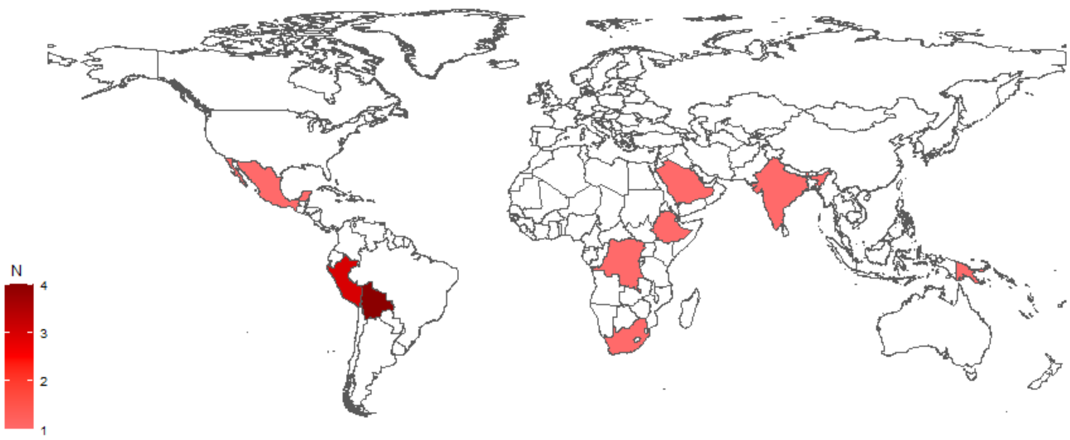

Supplementary material 4. Tables of the number of true and false positive, and true and false negative cases for hemoglobin vs. each comparator

Table S1. Results of diagnostic test accuracy Studies - hemoglobin vs Ferritin

Source	Patient population	Hemoglobin with factor correction	Cutoff of Reference Standard	Prevalence of anemia n (%)	Prevalence of iron deficiency n (%)	TP	TN	FP	FN
**A. Population: Adults over 18 years of age, non-pregnant.**									
Alkhaldy HY. et al. 2020	Female human medicine students aged 19 to 27 years (n = 200).	Yes	<15 ng/mL	41 (20.5)	105 (52.5)	35	6	89	70
		Yes	<20 ng/mL	41 (20.5)	126 (63)	38	3	71	88
		No (12.00 g/dL)	<15 ng/mL	13 (6.5)	116 (58)	NR	NR	NR	NR
Silubonde TM. et al. 2020	Healthy, non-pregnant women of African descent aged 18 to 25 years (n = 492).	No (12.45 g/dL)	<15 µg/L	192 (39)	178 (36.2)	100	92	228	72
		Yes	<15 µg/L*	192 (39)	185 (37.6)	105	87	220	80
		No (12.00 g/dL)	<15 µg/L*	91 (18.5)	185 (37.6)	62	29	278	123
		No (12.35 g/dL)	<15 µg/L*	183 (37.2)	185 (37.6)	103	80	227	82
		No (12.45 g/dL)	< 30 µg/L	192 (39)	254 (51.6)	131	61	177	123
Gebreegziabher T. et al. 2017	Non-pregnant women 18 - 52 years old.	Yes	≤15 µg/L*	43 (21.3)	36 (17.8)	10	33	133	26
Okumiya K 2016	Adult farmers and nomads	NR	≤12 ng/mL	59 (11.8)	116 (23.2)	46	13	371	70
Vaterlaws AL. et al. 1981	Adults in rural areas	Yes	<30 ug/L	10 (2.9)	64 (18.3)	6	4	262	78
**B. Population: Postpartum women**									
Villalpando S. et al. 2003	Postpartum women	Yes	≤12 µg/L	41 (62.1)	35 (53)	NR	NR	NR	NR
**C. Population: Children under 5 years of age**									
Gonzales, G.F. et al. 2020 and	infants 6 to 24 months	Yes	≤12ng/ml	126 (94.7)	35 (26.3)	NR	NR	NR	NR
		No (11.0 g/dL)	≤12ng/ml	15 (11.3)	35 (26.3)	NR	NR	NR	NR
Burke RM. Et al. 2018	Only infants with available data from all three blood draws.	Yes	≤12 µg/L*	204 (75.6)	151 (55.9)	125	79	40	26
Bahizire E. et al. 2017	Infants 6 to 59 months	Yes	≤12 ug/L	377 (46.9)	17 (2.1)	15	362	425	2
		Yes	<30 ug/L*	377 (46.9)	82 (10.2)	62	315	407	20
Burke RM. et al. 2017	1-5 months	Yes	≤ 12 µg/L	112 (70.0)	1 (0.5)	1	111	48	0
		Yes	≤ 12 µg/L*	112 (70.0)	1 (0.5)	1	111	48	0
	6-10 months	Yes	≤ 12 µg/L	121 (75.6)	64 (40.3)	56	65	30	9
		Yes	≤ 12 µg/L*	121 (75.6)	89 (55.5)	74	47	24	15
	10-19 months	Yes	≤ 12 µg/L	130 (81.3)	105 (65.5)	92	38	17	13
		Yes	≤ 12 µg/L*	130 (81.3)	127 (79.2)	108	22	12	18
**D. Population: Children from 6 to 10 years old**									
Miranda M. et al. 2015	Schoolchildren from 6 to 10 years of age in the peri-urban area.	Yes	<30ug/L*	35 (17.9)	38 (19.5)	34	1	116	44

TP: True Positives; TN: True Negatives; FP: False Positives; FN: False Negative; NR: Not reported

* Adjusted for inflammation

Table S2. Results of diagnostic test accuracy Studies - hemoglobin vs others marker of iron deficiency

Source	Patient population	Hemoglobin with factor correction	Cutoff of Reference Standard	Prevalence of anemia n (%)	Prevalence of iron deficiency n (%)	TP	TN	FP	FN
**A. hemoglobin vs sTFR**									
Silubonde TM. et al. 2020	Healthy, non-pregnant women of African descent aged 18-25 years (n = 492).	No (12.35 g/dL)	≥8,3 mg/L	183 (37.2)	204 (41.5)	NR	NR	NR	NR
Gebreegziabher T. et al. 2017	Non-pregnant women 18 - 52 years.	Yes	≥8,3 mg/L	43 (21.3)	10 (5)	3	40	152	7
Burke RM. et al. 2017	1-5 months	Yes	≥8,3 mg/L	112 (70.0)	2 (1.4)	1	111	47	1
		Yes	≥8,3 mg/L*	112 (70.0)	1 (0.6)	1	111	48	0
	6-10 months	Yes	≥8,3 mg/L	121 (75.6)	13 (8.4)	13	108	39	0
		Yes	≥8,3 mg/L*	121 (75.6)	12 (7.4)	11	110	39	0
	10-19 months	Yes	≥8,3 mg/L	130 (81.3)	39 (24.4)	36	94	28	2
		Yes	≥8,3 mg/L*	130 (81.3)	43 (26.8)	40	90	27	3
**B. hemoglobin vs Transferrin saturation**									
Gebreegziabher T. et al. 2017	Non-pregnant women over 18 years of age.	Yes	<15%	43 (21.3)	32 (15.8)	10	33	137	22
Villalpando S. et al. 2003	Postpartum women	Yes	≤16%	41 (62.1)	31 (47.0)	NR	NR	NR	NR
Moreno-Black G. et al. 1984	Women participating in the "Club de Madres" in Bolivia.	No (13.0 g/dL)	<15%	6 (3.9)	17 (11.1)	6	0	135	11
**B. hemoglobin vs TBI**									
Choque-Quispe BM. et al. 2020	Infants 6-59 months (n = 403).	No (11.0 g/dL)	< 0 (mg/kg)*	14 (3.5)	5 (2.5)	3	11	181	2
		Yes	< 0 (mg/kg)*	182 (45.2)	8 (4.1)	6	176	13	2
	Infants 6-35 months (n = 200).	No (11.0 g/dL)	< 0 (mg/kg)*	12 (6.0)	NR	NR	NR	NR	NR
		Yes	< 0 (mg/kg)*	122 (61.2)	NR	NR	NR	NR	NR
	Infants 36-59 months (n = 203).	No (11.0 g/dL)	< 0 (mg/kg)*	8 (3.9)	NR	NR	NR	NR	NR
		Yes	< 0 (mg/kg)*	138 (68.0)	NR	NR	NR	NR	NR
	Infants 36-59 months without inflammation (n = NR).	No (11.0 g/dL)	< 0 (mg/kg)*	NR	NR	NR	NR	NR	NR
		Yes	< 0 (mg/kg)*	NR	NR	NR	NR	NR	NR
Burke RM. et al. 2017	1-5 months	Yes	< 0 (mg/kg)	112 (70.0)	1 (0.6)	1	111	48	0
		Yes	< 0 (mg/kg)*	112 (70.0)	1 (0.6)	1	111	48	0
	6-10 months	Yes	< 0 (mg/kg)	121 (75.6)	44 (27.7)	41	80	36	3
		Yes	< 0 (mg/kg)*	121 (75.6)	62 (39.0)	57	64	33	6
	10-19 months	Yes	< 0 (mg/kg)	130 (81.3)	88 (54.8)	83	47	26	4
		Yes	< 0 (mg/kg)*	130 (81.3)	108 (67.3)	98	32	20	10
Gebreegziabher T. et al. 2017	Non-pregnant women 18 - 52 years.	Yes	< 0 (mg/kg)*	43 (21.3)	12 (5.9)	NR	NR	NR	NR
Alarcon-Yaquetto DE. et al. 2022	Male or female over 18 years of age, born in the city of Cusco and living permanently in the city. (n = 345).	No	< 0 (mg/kg)*	2 (0.6)	0 (0.0)	0	2	149	44
		Yes	< 0 (mg/kg)*	21 (6.1)	0 (0.0)	0	21	129	45
**D. hemoglobin vs others**									
Alarcon-Yaquetto DE. et al. 2022	Male or female over 18 years of age, born in the city of Cusco and living permanently in the city. (n = 345). Altitud = 3400 masl	No	TFR-F index < 1.0 and IL-6 < 50 pg/mL	2 (0.6)	12 (3.5)	1	1	147	46
		Yes	TFR-F index < 1.0 and IL-6 < 50 pg/mL	21 (6.1)	12 (3.5)	11	10	127	47
Gebreegziabher T. et al. 2017	Non-pregnant women 18 - 52 years old.	Yes	Plasma iron (<500 µg/L)	43 (21.3)	53 (26.2)	16	27	122	37
Villalpando S. et al. 2003	Postpartum women	Yes	serum iron (≤11 µmol/L)	41 (62.1)	35 (53.0)	NR	NR	NR	NR
		Yes	≥2 indexes (serum ferritin (≤12 g/L), transferrin saturation (≤16%), and MCV (≤80 fL))	41 (62.1)	26 (39.4)	24	17	23	2

sTFR: Soluble transferrin receptor; TBI: Total Body Iron; TP: True Positives; TN: True Negatives; FP: False Positives; FN: False Negative; NR: Not reported

* Adjusted for inflammation

Supplementary material 5. PRISMA-ScR Checklist

Preferred Reporting Items for Systematic reviews and Meta-Analyses extension for Scoping Reviews (PRISMA-ScR) Checklist

SECTION	ITEM	PRISMA-ScR CHECKLIST ITEM	REPORTED ON PAGE #
**TITLE**			
Title	1	Identify the report as a scoping review.	1
**ABSTRACT**			
Structured summary	2	Provide a structured summary that includes (as applicable): background, objectives, eligibility criteria, sources of evidence, charting methods, results, and conclusions that relate to the review questions and objectives.	2
**INTRODUCTION**			
Rationale	3	Describe the rationale for the review in the context of what is already known. Explain why the review questions/objectives lend themselves to a scoping review approach.	4
Objectives	4	Provide an explicit statement of the questions and objectives being addressed with reference to their key elements (e.g., population or participants, concepts, and context) or other relevant key elements used to conceptualize the review questions and/or objectives.	4
**METHODS**			
Protocol and registration	5	Indicate whether a review protocol exists; state if and where it can be accessed (e.g., a Web address); and if available, provide registration information, including the registration number.	5, not published.
Eligibility criteria	6	Specify characteristics of the sources of evidence used as eligibility criteria (e.g., years considered, language, and publication status), and provide a rationale.	5
Information sources*	7	Describe all information sources in the search (e.g., databases with dates of coverage and contact with authors to identify additional sources), as well as the date the most recent search was executed.	5
Search	8	Present the full electronic search strategy for at least 1 database, including any limits used, such that it could be repeated.	5 and supplementary material
Selection of sources of evidence†	9	State the process for selecting sources of evidence (i.e., screening and eligibility) included in the scoping review.	5-6
Data charting process‡	10	Describe the methods of charting data from the included sources of evidence (e.g., calibrated forms or forms that have been tested by the team before their use, and whether data charting was done independently or in duplicate) and any processes for obtaining and confirming data from investigators.	6
Data items	11	List and define all variables for which data were sought and any assumptions and simplifications made.	6
Critical appraisal of individual sources of evidence§	12	If done, provide a rationale for conducting a critical appraisal of included sources of evidence; describe the methods used and how this information was used in any data synthesis (if appropriate).	no critical appraisal.

SECTION	ITEM	PRISMA-ScR CHECKLIST ITEM	REPORTED ON PAGE #
Synthesis of results	13	Describe the methods of handling and summarizing the data that were charted.	6
**RESULTS**			
Selection of sources of evidence	14	Give numbers of sources of evidence screened, assessed for eligibility, and included in the review, with reasons for exclusions at each stage, ideally using a flow diagram.	7
Characteristics of sources of evidence	15	For each source of evidence, present characteristics for which data were charted and provide the citations.	8-10
Critical appraisal within sources of evidence	16	If done, present data on critical appraisal of included sources of evidence (see item 12).	-
Results of individual sources of evidence	17	For each included source of evidence, present the relevant data that were charted that relate to the review questions and objectives.	11-15
Synthesis of results	18	Summarize and/or present the charting results as they relate to the review questions and objectives.	11-15
**DISCUSSION**			
Summary of evidence	19	Summarize the main results (including an overview of concepts, themes, and types of evidence available), link to the review questions and objectives, and consider the relevance to key groups.	16
Limitations	20	Discuss the limitations of the scoping review process.	18-19
Conclusions	21	Provide a general interpretation of the results with respect to the review questions and objectives, as well as potential implications and/or next steps.	19
**FUNDING**			
Funding	22	Describe sources of funding for the included sources of evidence, as well as sources of funding for the scoping review. Describe the role of the funders of the scoping review.	7

JBI = Joanna Briggs Institute; PRISMA-ScR = Preferred Reporting Items for Systematic reviews and Meta-Analyses extension for Scoping Reviews.

* Where *sources of evidence* (see second footnote) are compiled from, such as bibliographic databases, social media platforms, and Web sites.

† A more inclusive/heterogeneous term used to account for the different types of evidence or data sources (e.g., quantitative and/or qualitative research, expert opinion, and policy documents) that may be eligible in a scoping review as opposed to only studies. This is not to be confused with *information sources* (see first footnote).

‡ The frameworks by Arksey and O'Malley (6) and Levac and colleagues (7) and the JBI guidance (4, 5) refer to the process of data extraction in a scoping review as data charting.

§ The process of systematically examining research evidence to assess its validity, results, and relevance before using it to inform a decision. This term is used for items 12 and 19 instead of "risk of bias" (which is more applicable to systematic reviews of interventions) to include and acknowledge the various sources of evidence that may be used in a scoping review (e.g., quantitative and/or qualitative research, expert opinion, and policy document).

From: Tricco AC, Lillie E, Zarin W, O'Brien KK, Colquhoun H, Levac D, et al. PRISMA Extension for Scoping Reviews (PRISMAScR): Checklist and Explanation. Ann Intern Med. 2018;169:467–473. doi: 10.7326/M18-0850.
